# Parry Romberg syndrome: A case report

**DOI:** 10.1016/j.radcr.2024.09.029

**Published:** 2024-09-24

**Authors:** Yaa Achiaa Afreh, Kwasi Adjepong Twum, Adu Tutu Amankwa, Godwin Ashley, Kwasi Ankomah, Obed Kojo Otoo

**Affiliations:** a Radiology Directorate, Komfo Anokye Teaching Hospital (KATH). P.O. Box 1934, Kumasi Ghana; b Department of Radiology, School of Medicine and Dentistry, Kwame Nkrumah University of Science and Technology (KNUST), Private Mail Bag, University Post Office, Kumasi Ghana

**Keywords:** Parry Romberg syndrome, Hemifacial atrophy, Computed tomography

## Abstract

Parry Romberg syndrome (PRS) is a rare self-limiting disease, typically occurring in children and young adults, that causes slow progressive atrophy of one-half of the face. It primarily affects the subcutaneous tissue and skin with some cases exhibiting deeper extension to glandular, osseous and muscular structures. Neurologic and ocular involvement is variable. Neuroimaging with computed tomography (CT) scan aids in demonstrating radiological features, assessing disease severity, and detecting neurological and ocular complications. We present a severe case of PRS in a 25-year-old female with right-sided facial asymmetry, diagnosed based on medical history, clinical examination and head CT scan findings.

## Introduction

Parry-Romberg syndrome (PRS), also known as progressive hemifacial atrophy, is a rare developmental disorder with a prevalence of at least 1 in 700,000 births [1]. It was initially described by Dr. Caleb Hillier Parry in 1825 and further detailed by Dr. Moritz Heinrich Romberg in 1846 [2,3]. The current nomenclature, "progressive hemifacial atrophy," was however coined in 1871 by Eulenberg [4]. PRS is characterized by unilateral atrophy of facial tissues, primarily affecting subcutaneous fat but also exhibiting variability in the involvement of skin, muscle, cartilage, and bone.

Manifesting in childhood, usually within the initial 2 decades of life, PRS exhibits a higher prevalence in females compared to males [1,5,6] and the left side of the face is more frequently affected. After disease onset, the condition follows a gradual progression that eventually reaches a stable or “burnt out” phase [7]. Although not life-threatening, the resultant craniofacial asymmetry causes aesthetic discomfort. Additionally, patients may experience neurological and ophthalmological complications, including seizures, trigeminal neuralgia, migraines and enophthalmos [8,9].

While PRS is often diagnosed clinically, radiological investigations are imperative to assess the extent of soft tissue or bony atrophy, identify intracranial involvement and monitor disease progression [10].

We present a case of a 25-year-old female diagnosed with Parry Romberg syndrome.

## Case presentation

A 25-year-old female sought evaluation at the Radiology Directorate of the Komfo Anokye Teaching Hospital in Ghana, reporting concerns of facial asymmetry and hyperpigmentation on the right side of her face. A shallow depression was initially noticed on the right malar region at the age of 3, which progressively advanced over the last 20 years. Over the past 2 years, the symptoms remained stable. The patient reported no associated symptoms and her past medical and family history yielded no significant findings.

**Fig. 1:**
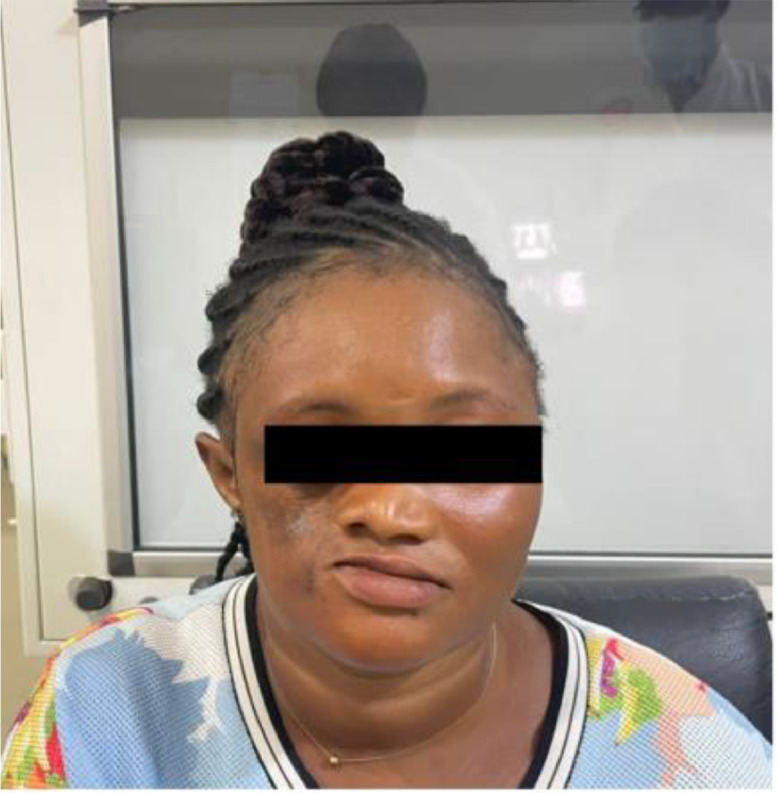
Photograph of patient showing right hemifacial atrophy predominant at the maxillary region, deviation of the mouth and nose to the right and hyperpigmentation of the overlying skin.

Upon physical examination, noticeable right-sided facial atrophy was observed, particularly involving the maxillary region, accompanied by hyperpigmentation of the overlying skin (Fig. 1). The examination also revealed a discernible loss of underlying fat and muscles, resulting in a sunken appearance. Additionally, there was reduction in the size of the right upper lip and right nostril, along with a deviation of the corner of the mouth and nose to the right (Fig. 1). Intraoral examination indicated no tongue atrophy, although there was reduction in the size of the right upper teeth. No alopecia was noted, and neurological and ophthalmological examinations, as well as blood investigations, showed no remarkable findings.

**Fig. 2:**
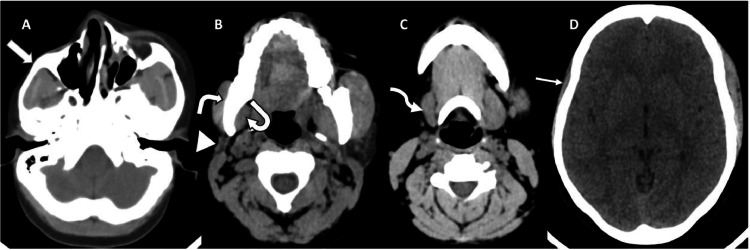
(A-D) Axial head CT scan (soft tissue window) showing thinning of the subcutaneous fat and skin over the right malar region (*block arrow*) (**A**) as well as atrophy of the right parotid gland (*arrowhead*) (**B**), right submandibular gland (*squiggle arrow*) (**C**), right masseter muscle (*white bent arrow*) (**B**), right medial pterygoid muscle (*curved arrow*) (**B**) and right temporalis muscle (*straight arrow*) (D).

**Fig. 3:**
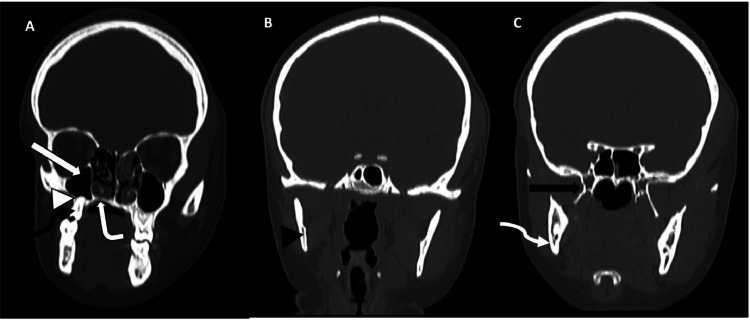
(**A-C**) Coronal reformatted CT scans of the head (bone window) shows reduction in the sizes of the palatine process (*white bent arrow*) and the alveolar process (*white arrowhead*) of the right maxilla with a smaller right maxillary sinus (*white block arrow*) and first right upper molar (*black squiggle arrow*) (**A**). There is also reduction in the size of the right mandibular ramus (*black arrowhead*) (**B**), the right mandibular angle (*white squiggle arrow*), the body, medial and lateral processes of the right pterygoid bone (*black block arrow*) (**C**).

**Fig. 4:**
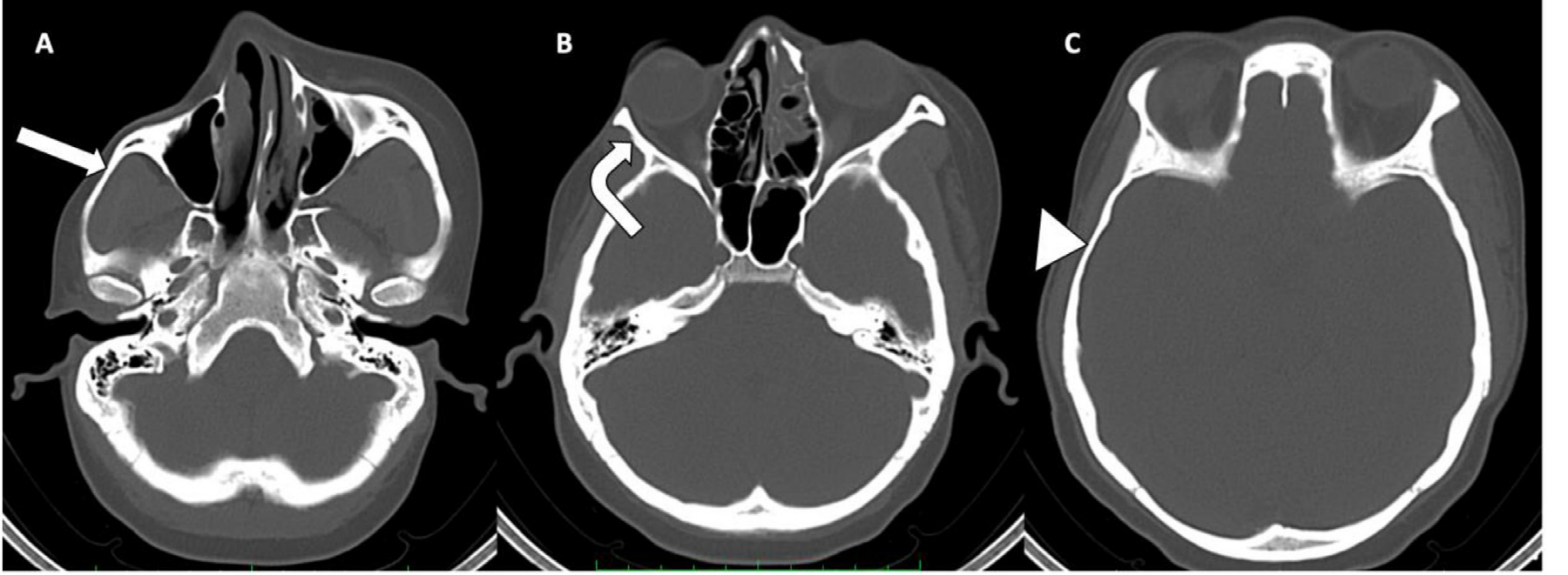
(**A-C**) Axial CT scans of the head (bone window) showing atrophy of the right zygoma and zygomatic process (*white bloack arrow*) (**A**), the right greater wing of sphenoid bone (*white bent arrow*) (**B**) and squamous part of the right temporal bone (*white arrowhead*) (**C**).

**Fig. 5:**
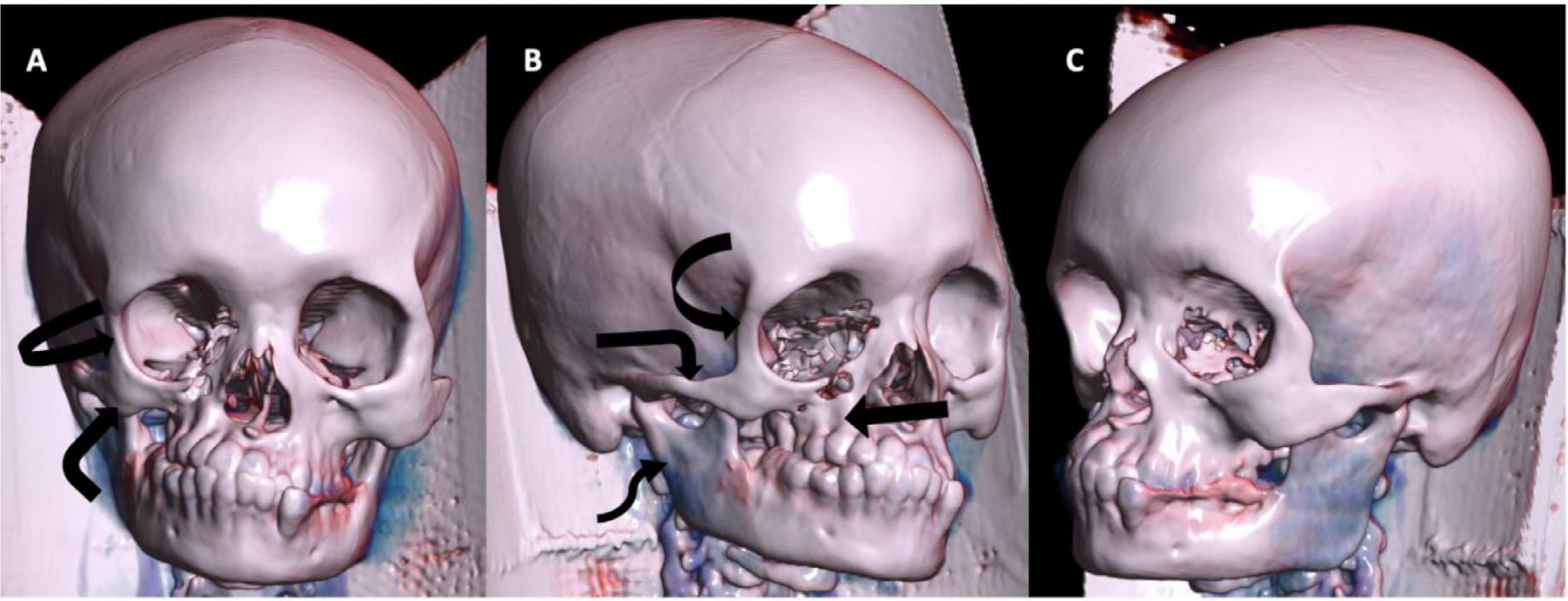
(**A-C**) Volume rendered 3D images with frontal (**A**), right lateral (**B**) and left lateral (**C**) views of the head showing atrophy of the right maxilla (*block arrow*), right zygoma/zygomatic arch (*bent arrow*), right hemimandible (*squiggle arrow*) and greater wing of the right sphenoid bone (*curved arrow*) (**A-B**). Left lateral view (**C**) included for comparison.

Computed tomography (CT) of the head unveiled atrophy on the right side of the face, involving the subcutaneous fat, muscles of mastication (masseter, temporalis, and pterygoid muscles), parotid and submandibular glands (Fig. 2 A-D). Bony involvement was evident, with atrophy affecting the right zygoma, right sphenoid bone, right side of the maxilla and mandible, as well as the squamous part of the right temporal bone (Figs. 3, Fig. 4, Fig. 5 A-C). Reduction in the sizes of the right upper premolars and molars was also noted (Fig. 3 A). Importantly, the brain parenchyma was normal.

Upon integrating all imaging findings with the patient's history and physical examination, a conclusive diagnosis of Parry Romberg syndrome was established.

## Discussion

PRS is an infrequent, sporadic, neurocutaneous disorder characterized by progressive tissue atrophy on 1 side of the face. It exhibits a female predilection, with a male-female ratio of 1:2.2[6], and a tendency for the left side of the face to be more commonly affected [10,11,12].

The etiopathogenesis of PRS remains poorly understood leading to various proposed theories such as autoimmune dysfunction, trauma, infectious etiology, vascular abnormalities and dysfunction of the sympathetic cervical ganglia [5,7,13]. Current evidence strongly supports PRS as an autoimmune disorder, indicated by the presence of coexisting autoimmune conditions like systemic lupus erythematosus and rheumatoid arthritis, as well as clinical improvement with immunosuppressive therapy during active disease [5,7].

It manifests typically within the first 2 decades of life, commonly between 5 and 15 years, with a median onset age of 10 years. PRS then follows a progressive and insidious course spanning 2-20 years postonset, eventually stabilizing [1,7,12,14]. While late occurrences of the disease are uncommon, they have been reported [1,10]. Additionally, an earlier onset and a more prolonged duration of PRS are associated with more severe manifestations [10,13].

Clinically, patients present with varying degrees of atrophy primarily affecting subcutaneous fat and skin and progressing to muscular, osseous and glandular structures. The damage is mostly confined to the distribution of the trigeminal nerve and is classified as mild, moderate and severe, depending on the extent of hemifacial atrophy. In mild cases, atrophy is confined to the skin and subcutaneous tissue, limited to a single sensory branch of the trigeminal nerve. Moderate disease involves atrophy extending to 2 branches of the trigeminal nerve, while severe disease encompasses atrophy affecting all 3 branches of the trigeminal nerve or any bone involvement [15]. Atrophy typically begins at the maxillary or periorbital region but may extend to other facial areas, with accompanying changes in pigmentation of the overlying skin [13,14]. Although often limited to one-half of the face, bilateral facial involvement has been reported in 2% and 7.4% of cases by Stone et al. and Tollefson et al., respectively [12,16]. Ipsilateral body or limb involvement is exceedingly rare [9].

Our patient presented with a severe form of PRS characterized by atrophy affecting the subcutaneous tissue, muscles, bones, and salivary glands. This severe manifestation was not unexpected, given the remarkably early onset of symptoms and the prolonged duration of the disease. The observed dental involvement aligns with the early age of onset, serving as a valuable indicator in determining the age of onset, particularly in cases with unclear timelines [8]. Notably, the atypical aspect of our case was the involvement of the right side of the face, deviating from the more commonly observed left-sided affection in PRS.

Neurological complications occur in 15%-20% of cases, presenting as headaches, seizures, trigeminal neuritis, facial paresthesia, cranial nerve dysfunction and fixed focal neurological deficits. Headaches and seizures, often refractory to treatment, are the most common [5]. Ophthalmologic symptoms, observed in 10-35% of patients, typically involve the ipsilateral orbit [7]. Enophthalmos due to the retrobulbar fat atrophy is frequently described. Less commonly encountered orbital manifestations include uveitis, retinal changes and restrictive strabismus [5,7]. It is noteworthy that in our patient, no neurological or ophthalmological complications were observed.

Neuroimaging findings in PRS are reported to be abnormal in 20%-50% of patients, with those exhibiting neurological symptoms more likely to show positive findings on brain imaging [13]. These abnormalities are typically ipsilateral to the affected side of the face but may also be bilateral, remaining generally stable on serial imaging and inconsistently associated with neurological manifestations [13,17]. Common findings on CT or magnetic resonance imaging (MRI) include focal or hemispheric cerebral atrophy, subcortical calcifications, encephalomalacia and white matter lesions. Less frequently reported abnormalities involve ventricular dilatation, corpus callosal infarcts, microhemorrhages and vascular stenoses or aneurysms [7,17]. Our case, however, displayed normal brain imaging with no abnormalities evident on the head CT scan.

The differential diagnosis for PRS includes en coupe de sabre (ECDS), hemifacial microsomia, Goldenhar syndrome and Rasmussen encephalitis. ECDS, a variant of linear scleroderma, shares similar epidemiological and clinical features with PRS. These conditions often coexist and are believed to be entities on the same spectrum of disease, with reported cases of ECDS converting to PRS [5,8,13]. Distinguishing between these 2 conditions can be challenging. However, bony, muscular, and cutaneous atrophy favor a diagnosis of PRS, while predominant involvement of the fronto-parietal region with no extension below the eyebrow and cutaneous sclerosis are more characteristic of ECDS [5,7,13]. Hemifacial microsomia and Goldenhar syndrome are congenital, nonprogressive disorders. Rasmussen encephalitis is an acquired inflammatory autoimmune disorder that manifests in the pediatric population with intractable partial epileptic seizures and progressive neurological deficits. While similar brain imaging findings may be observed in Rasmussen encephalitis and PRS, a history of refractory epileptic seizures with the absence of facial asymmetry should favor a diagnosis of Rasmussen encephalitis [13]. Other conditions that can clinically mimic PRS include fat necrosis from connective tissue diseases, infectious causes or traumatic injuries [5].

Treatment of PRS can be challenging, as there are currently no standard treatment guidelines in place. The primary objective of treatment is to impede the progression of the disease using immunosuppressive agents and to manage seizures through the administration of anticonvulsants. The success of these therapeutic measures however varies among individuals [5,7]. Surgical interventions to address cosmetic disfigurement are typically employed once the disease has stabilized. This approach necessitates a multidisciplinary strategy and may involve procedures such as autologous fat grafts, muscle flap grafts, silicone injections, bone augmentation, and orthodontic rehabilitation [7,13]. Additionally, patients benefit from psychological support, highlighting the importance of addressing the emotional and psychological aspects of living with PRS [18].

In the case of our patient, counseling was provided regarding potential surgical treatment options for aesthetic management and follow-up is planned to monitor overall patient progress.

## Conclusion

PRS is an uncommon condition characterized by hemifacial atrophy, often accompanied by neurological and ophthalmological symptoms. A precise clinical diagnosis necessitates comprehensive knowledge of the condition, detailed review of the patient's medical history, thorough physical examination and neuroimaging. The classic features highlighted in our case report will contribute significantly to enhancing understanding of this disease and aid in the exclusion of potential differential diagnoses.

## Patient consent

A written informed consent relating to use of patient's medical history and radiological images was obtained after careful explanation to the patient’ that their anonymized images and clinical history will be used for publication in a scientific journal.

## Appendix Supplementary materials

Image, video 1

Image, video 2
